# Antimicrobial Natural Product Berberine Is Efficacious for the Treatment of Atrial Fibrillation

**DOI:** 10.1155/2017/3146791

**Published:** 2017-12-17

**Authors:** Hongchao Zheng, Fu Zhu, Peizhi Miao, Zhenzhen Mao, Damian P. Redfearn, Richard Y. Cao

**Affiliations:** ^1^Department of Cardiology, Shanghai Xuhui Central Hospital/Zhongshan-Xuhui Hospital, Fudan University, Shanghai Clinical Research Center, Chinese Academy of Sciences, 966 Middle Huaihai Road, Shanghai 200031, China; ^2^Department of Medicine, Kingston General Hospital, Queen's University, 76 Stuart Street, Kingston, ON, Canada K7L 2V7

## Abstract

The purpose of this study is to test the efficacy of bioactive natural product berberine in the treatment of patients with atrial fibrillation (AF). Data of 45 paroxysmal AF patients treated with berberine and 43 age, gender, New York Heart Association functional classification score, and concomitant cardiovascular disease matched patients treated with amiodarone were analyzed retrospectively to examine conversion rate, average conversion time, average heart rate, and echocardiographic parameters. There was no statistical difference between berberine and amiodarone on conversion rate or echocardiographic parameters. Berberine treatment showed a significantly longer average time to conversion and higher heart rate during sinus rhythm (SR) than amiodarone. Echocardiographic parameters showed that E/A ratio and left atrial diameter were significantly improved after 6- and 12-month berberine treatment, but only E/A ratio improved significantly at the same time points after amiodarone treatment. This is the first report to specifically compare efficacy of berberine and amiodarone in the treatment of patients with AF. We find that berberine and amiodarone are equally effective for conversion of AF and maintenance of normal SR.

## 1. Introduction

Bioactive natural products have been reported to exhibit antioxidant, antimicrobial, antiviral, anticancer, and anti-inflammatory activities [1]. Berberine, originally used as an antimicrobial medication for the treatment of diarrhea [2], is a natural product isoquinoline alkaloid isolated from Chinese medicinal plant* Coptis chinensis* [3]. Berberine's potential antiarrhythmic properties were first explored in a cohort of 50 patients with ventricular tachyarrhythmia conducted in Shanghai Xuhui Central Hospital by Dr. Huang et al. in 1985 [4]. Huang's team further published results of antiarrhythmic mechanisms of berberine in a canine model in 1992 [5]. Later Dr. Liu and Rao reported using berberine as an antiarrhythmic drug to treat 28 patients with ventricular premature beats in 1994 [6]. These early data showed potential for this agent as an antiarrhythmic with only minor gastrointestinal side effects but without comparing to other antiarrhythmics. The purpose of this study was to test the efficacy of berberine in the treatment of patients with atrial fibrillation (AF) in comparison with amiodarone, a frequently prescribed and widely used Vaughn-Williams class III agent efficacious in converting AF to sinus rhythm (SR) [7, 8].

## 2. Methods

This study was approved by the Ethics Committee of Shanghai Xuhui Central Hospital (Approval number 2013-12) with ethical standards of the Helsinki Declaration of 1964. To extend indication of berberine for AF, consecutive patients randomly received berberine or amiodarone treatment between January 2013 and March 2014 based on attending physician discretion. We conducted a retrospective cohort study in this patient database to compare the efficacy of berberine and amiodarone in the treatment of AF. Eligibility for inclusion included symptomatic paroxysmal AF (PAF) patients diagnosed by electrocardiogram (ECG) with history of 6 months–2 years, presenting to the emergency room with symptomatic AF and onset time of 3 hours–7 days, and they were aged between 20 and 80 years old and had oral administration of either berberine or amiodarone for 12 months. Patients were excluded from the analysis if they had ECG evidence of second-degree atrioventricular block, a diagnosis of sick sinus syndrome, thyroid dysfunction, severe lung disease, severe electrolyte disorder, severe hepatic disease, renal insufficiency, and stroke or any reversible cause of AF. Berberine and amiodarone treated patients were matched by age, gender, New York Heart Association (NYHA) functional classification score, and concomitant cardiovascular disease (CVD).

### 2.1. Study Procedure

**Figure 1:**
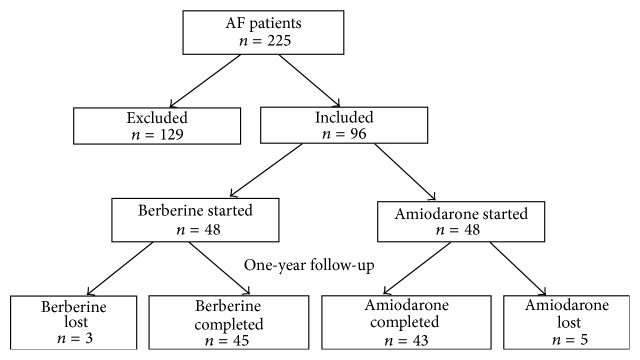
*Flow chart of patient selection.* From a database of 225 patients between January 2013 and March 2014, 96 met the inclusion criteria and 129 were excluded because of noneligibility. Data from 48 atrial fibrillation (AF) patients treated with berberine and 48 matched AF patients treated with amiodarone were included. Follow-up at one year was complete in 45 and 43 of berberine and amiodarone treated patients, respectively. Three patients were lost in berberine group and 5 patients were lost in amiodarone group.

Flowchart of patient selection was shown in Figure 1. From a database of 225 patients, 96 met the inclusion criteria and 129 were excluded because of noneligibility. Data from 48 AF patients treated with berberine and 48 matched AF patients treated with amiodarone were included. Follow-up at one year was complete in 45 and 43 of berberine and amiodarone treated patients, respectively. Three patients were lost in berberine group and 5 patients were lost in amiodarone group. Antiarrhythmic drugs were initiated during AF. No electrical cardioversion was applied to these PAF patients. Study schedule was shown in Supplementary Figure 1. The oral administration dose of berberine was minimal at 1.2 g/day (0.3 g qid) and maximal at 2.0 g/day (0.5 g qid), and the average dose is of 1.3 g/day for 1 year. Amiodarone was taken orally with an initial dose of 0.6 g/day (0.2 g tid) in the first week and then 0.4 g/day (0.2 g bid) in the second week followed by 0.2 g/day (0.2 g qd) in the third week and lasted for 11.5 months. Surface ECG was performed for each patient every week in the first month followed by once a month in the rest of the year. Echocardiography and blood test were conducted and adverse events were recorded at the follow-up time points of 1, 3, 6, and 12 months.

### 2.2. Outcome Assessment

The primary outcome was presence of SR after antiarrhythmic treatment with berberine or amiodarone at 1, 3, 6, and 12 months. The secondary outcome included average time from onset of therapy to conversion of AF to SR and heart rate in SR after successful treatment. The third outcome consisted of Doppler echocardiographic parameters E/A ratio (ratio of the early to late ventricular filling velocities) and left atrial diameter (LAD) measured at the follow-up time points of 1, 3, 6, and 12 months. The fourth endpoints were adverse events.

### 2.3. Statistical Analysis

The primary outcome time-to-event (conversion to SR) was compared between berberine and amiodarone groups by the Kaplan-Meier method. The statistically significant difference of two groups was analyzed by Log rank test. To adjust for potential confounding, the difference between two groups was further analyzed by Cox proportional hazard model. In the model analysis, we adjusted for the effects of age, sex, AF duration, frequency, history, and smoking status. Other endpoints between groups were compared by ANCOVA analysis with baseline value of the dependent variable as covariates.

## 3. Results

Baseline characteristics of the 88 patients followed to one year including age, gender, PAF history, smoking status, biochemical measurements, NYHA Functional Classification, comorbidities, and concomitant therapies were summarized in Table 1; there was no significant difference between berberine and amiodarone groups.

### 3.1. Proportion in SR

**Figure 2:**
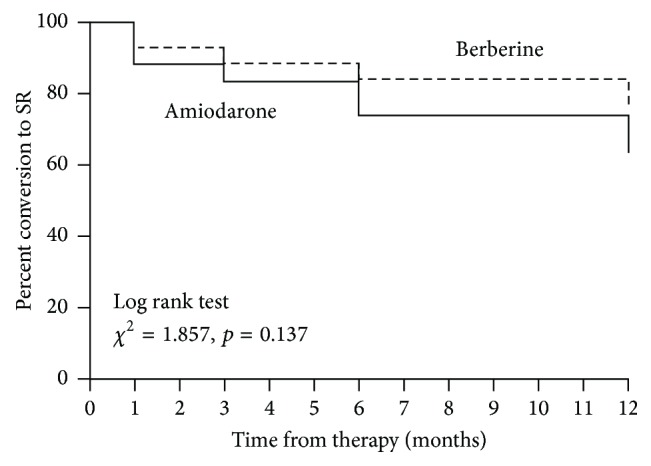
*Percent conversion to sinus rhythm.* Percentages of patient conversion of atrial fibrillation (AF) to sinus rhythm (SR) were plotted by Kaplan-Meier curve after 1-, 3-, 6-, and 12-month berberine or amiodarone treatment. There was no significant difference between two groups at any time points by Log rank test (*x*^2^ = 1.857, *p* = 0.137). Berberine: dashed line; amiodarone: solid line.

The number of patients conversion to SR at time points of 1, 3, 6, and 12 months was summarized in Table 2, while the percent conversion to SR was plotted by Kaplan-Meier curve in Figure 2. A trend toward a larger proportion of the berberine treated group in SR at 12 months was noted; however, when comparted with amiodarone, this was not statistically significant when analyzed by the Cox proportional hazard model after adjusting for the effects of age, gender, AF duration, frequency, history, and smoking status (*x*^2^ = 3.156, *p* = 0.0756). There was a high proportion of SR in both groups at 1 month but noticeable attrition over time with a slight statistical difference between 1-month and 12-month time points in the amiodarone treatment group (*p* = 0.04).

### 3.2. Time to Conversion and Heart Rate

Patients were closely monitored during the first day of berberine or amiodarone oral therapy. The average time to achieve SR in the patients given berberine was significantly longer than that in amiodarone group (356 ± 135 versus 211 ± 126 min,* p* < 0.01). The average heart rate during SR measured at 1-month follow-up in berberine group was significantly higher than that in amiodarone group (83.5 ± 11.6 versus 69.3 ± 12.5/min,* p* < 0.01).

### 3.3. Echocardiographic Parameters

Measurements of echocardiographic parameters E/A ratio and LAD were summarized in Table 3. No statistical difference in the analyzed echocardiographic parameters E/A ratio and LAD was detected between berberine and amiodarone groups before treatment and 1, 3, 6, and 12 months after treatment. Nevertheless, the ANCOVA analysis did reveal a significant effect for time, *F*  (4, 344) = 14.32,* p *< 0.001. In the berberine group, post hoc contrasts found that the values of E/A at 6- (*p *< 0.05) and 12-month time points (*p *< 0.01) were significantly improved than the measures observed before treatment. The LAD measures showed similar results (*p *< 0.05 at both 6- and 12-month time points). In the amiodarone group, only E/A ratio improved after 6- and 12-month (*p *< 0.05) treatment. LAD did not change statistically after amiodarone treatment at any follow-up time points.

### 3.4. Adverse Events

Five from the initial 48 amiodarone treated PAF patients did not complete 1-year treatment, in which 2 had dysthyroidism. Minor side effects were observed in 5 of the 43 patients in amiodarone group: 2 had transient sinus bradycardia, 2 had nausea, and 1 had dizziness. After reducing dose or withdrawing medication for several days, these reactions disappeared. Meanwhile, there were no severe adverse events recorded in the 45 patients who completed 12-month berberine treatment. But 3 patients from berberine group were lost during the study whose possible adverse events were not recorded. Only data of patients who were compliant were collected for this study.

## 4. Discussion

This retrospective study provides some support for an antiarrhythmic effect of berberine in patients with PAF. Although the primary outcome of proportion of SR did not show a statistical difference between groups, our data appear to demonstrate at least equivalence with the most effective antiarrhythmic medication available in clinical studies [7–9] and improved echocardiographic parameters for both E/A ratio and LAD after berberine treatment, but only E/A ratio improved in amiodarone group.

Amiodarone is not only the most commonly used antiarrhythmic medication in Chinese AF patients [10], but also the most-often-prescribed in developed countries, accounting for 34.5% of prescriptions in Europe and 32.8% in North America [8]. Nevertheless, long-term high dose administration of amiodarone is associated with both cardiac and noncardiac adverse effects, including sinus bradycardia, decreased blood pressure, pulmonary toxicity, liver toxicity, optic neuropathy, skin discoloration, and thyroid dysfunction [11]. We observed amiodarone side effects such as minor nausea and dizziness, transient sinus bradycardia, and dysthyroidism during this study. Therefore, finding alternative antiarrhythmic drugs with similar or better efficacy but less side effects is extremely desirable.

Berberine has been reported to be effective for* E. coli* diarrhea with a low dosage of 0.4 g/day but has no efficacy for* Vibrio cholera* even with a high dosage of 1.2 g/day [2]. Our data demonstrated that berberine with a minimal oral administration dose of 1.2 g/day had a nonsignificant trend toward higher proportion of SR during the one-year follow-up when compared with amiodarone (Figure 2). However, berberine needed a significantly longer time to reach SR (356 ± 135 versus 211 ± 126 min,* p *< 0.01) and a higher heart rate in SR (83.5 ± 11.6 versus 69.3 ± 12.5/min,* p *< 0.01) in comparison to amiodarone after successful treatment. These phenomena imply that berberine is efficacious at rhythm control but may not result in any rate control during AF, judged by the average heart rate in SR. The possible explanation of the slow effect of berberine is that its antiarrhythmic mechanism is via a reduction in potassium current in a concentration-dependent manner, which takes a longer time [5].

Although berberine and amiodarone were statistically equally effective in maintenance of SR after 1, 3, 6, and 12 months of treatment, evidence of reverse atrial remodelling was only statistically significant for berberine with reduction in LAD size demonstrated at 6 months (*p* < 0.05) and 12 months (*p* < 0.01) supporting a potent antiarrhythmic effect and true reduction in burden of AF in this cohort. In addition, there were no serious adverse events recorded during the 12-month berberine treatment. This result was consistent with another clinical trial with a higher berberine daily dose of 1.5 g for 16 weeks, in which minor adverse events such as anorexia, upset stomach, diarrhea, and constipation were recorded, but no serious adverse events such as heart failure, bone fractures, and liver toxicity were observed [12]. Nevertheless, our retrospective setting limited the choice of variables to record for analysis. Three lost patients from berberine group may have adverse events but were not recorded. Included patients who completed 1-year berberine treatment were less likely to have severe adverse effects.

In summary, our results are novel as no previous clinical studies have specifically compared the efficacy of berberine and amiodarone for the treatment of AF. We find that berberine and amiodarone are equally effective at maintenance of SR. The health beneficial effect of berberine is confirmed and the clinical application of berberine is promising. The knowledge of adverse effects with berberine is limited; however monitoring the safety of berberine in the high dose range is important. Therefore, further prospective studies are needed to evaluate the long-term efficacy and side effect profile of berberine in a contemporary AF population.

## Figures and Tables

**Table 1:** Characteristics of PAF patients by berberine and amiodarone groups.

Category	Berberine *N* = 45	Amiodarone *N* = 43	Significance
Age (year)	65 ± 11	63 ± 12	NS
Male	28 (62.2%)	27 (62.8%)	NS
Duration (hour)	48	46	NS
Frequency (time/month)	5.5	6.3	NS
History (month)	9.5	9.3	NS
Smoking status			
Never smoking	22 (48.9%)	20 (46.6%)	NS
Smoking < 5 year	8 (17.8%)	9 (21%)	NS
Smoking ≥ 5 year	15 (33.4%)	14 (32.6%)	NS
K (mmol/L)	4.1	4.2	NS
Mg (mmol/L)	1.0	1.1	NS
TSH (u/ml)	1.5	1.4	NS
ALT (u/ml)	17	18	NS
AST (u/ml)	16	18	
CR (*µ*mol/L)	76	74	NS
PT (sec)	12.2	12.8	NS
INR	0.92	0.95	NS
NYHA classification			
ΙΙ	31	29	NS
ΙΙΙ	14	14	NS
Concomitant CVD			
CAD	39	36	NS
DCM	6	7	NS
HTN	41	40	NS
HF	2	3	NS
Concomitant medication			
Aspirin	21	20	NS
Nitrates	22	21	NS
ACE inhibitors	41	41	NS
Statins	21	20	NS

*Notes.* ACE, angiotensin-converting enzyme; ALT, alanine transaminase; AST, aspartate transaminase; CAD, coronary artery disease; CR, creatinine; CVD, cardiovascular disease; DCM, dilated cardiomyopathy; HF, heart failure; HTN, hypertension; INR, international normalized ratio; K, potassium; Mg, magnesium; NS, nonsignificant between groups; NYHA, New York Heart Association; PT, prothrombin time; TSH, thyroid-stimulating hormone.

**Table 2:** Reversion rates of sinus rhythm in berberine and amiodarone groups.

Group	*N*	Follow-up time			
		1 month	3 months	6 months	12 months
Berberine	45	42 (93.3%)	40 (88.8%)	38 (84.4%)	35 (77.7%)
Amiodarone	43	37 (86.1%)	35 (81.3%)	31 (72%)	28 (65.1%)^*∗*
Significance		NS	NS	NS	NS

*Notes*.^*∗*^*p* < 0.05 1 month versus 12 months; NS, nonsignificant between two groups.

**Table 3:** Echocardiographic parameter improvement over time.

Group	Parameter	Before treatment (baseline)	After treatment			
			1 month	3 months	6 months	12 months
Berberine	E/A	0.94 ± 0.37	0.96 ± 0.36	1.05 ± 0.37	1.15 ± 0.39^*∗*^	1.26 ± 0.35^#^
Amiodarone	E/A	0.95 ± 0.31	0.97 ± 0.30	1.04 ± 0.34	1.14 ± 0.33^*∗*^	1.25 ± 0.32^*∗*^
Significance		NS	NS	NS	NS	NS
Berberine	LAD (mm)	36.9 ± 13.5	35.5 ± 12.5	33.6 ± 12.5	30.8 ± 13.1^*∗*^	30.5 ± 13.6^*∗*^
Amiodarone	LAD (mm)	35.5 ± 13.9	34.9 ± 12.9	32.8 ± 12.1	31.4 ± 13.9	30.1 ± 13.8
Significance		NS	NS	NS	NS	NS

*Notes.* ^*∗*^ *p* < 0.05 baseline versus 6 months or 12 months; ^#^*p* < 0.01 baseline versus 12 months; E/A, ratio of the early to late ventricular filling velocities; LAD, left atrial diameter; NS, nonsignificant difference between two groups.

## Supplementary Materials

Supplementary Materials Supplementary Figure  1. Study procedure. A. Berberine was administrated orally at minimal dose of 1.2 g/day (0.3 g qid) and maximal dose of 2.0 g/day (0.5 g qid) with an average dose of 1.3 g/day for 1 year. B. Amiodarone was taken orally with an initial dose of 0.6 g/day (0.2 g tid) in the first week and then 0.4 g/day (0.2 g bid) in the second week and followed by 0.2 g/day (0.2 g qd) in the third week and for the rest of the year. Follow-up time points were 1, 3, 6, and 12 months after treatment.
