# Supplementary material for: Antimicrobial Natural Product Berberine Is Efficacious for the Treatment of Atrial Fibrillation
Source: Biomed Res Int. 2017 Dec 17;2017:3146791. doi: 10.1155/2017/3146791 (PMC5748134; doi:10.1155/2017/3146791)
Supplement: Supplementary Materials — Supplementary Figure 1. Study procedure. A. Berberine was administrated orally at minimal dose of 1.2 g/day (0.3 g qid) and maximal dose of 2.0 g/day (0.5 g qid) with an average dose of 1.3 g/day for 1 year. B. Amiodarone was taken orally with an initial dose of 0.6 g/day (0.2 g tid) in the first week and then 0.4 g/day (0.2 g bid) in the second week and followed by 0.2 g/day (0.2 g qd) in the third week and for the rest of the year. Follow-up time points were 1, 3, 6, and 12 months after treatment. [file 3146791.f1.pdf]

Supplementary Figure 1

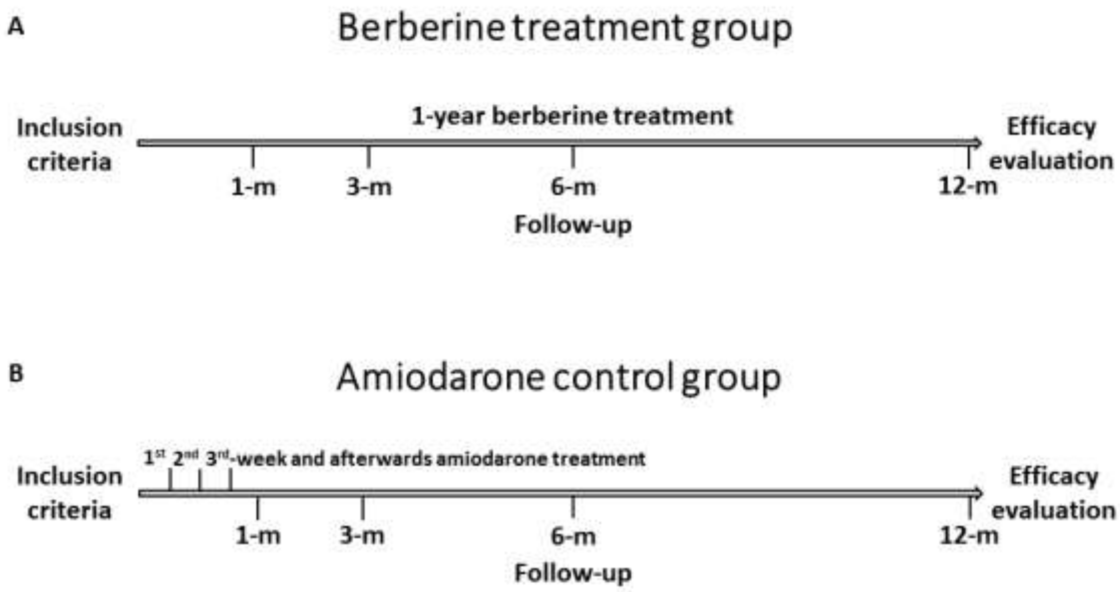

Supplementary Figure 1. Study procedure

A. Berberine was administrated orally at minimal dose of 1.2g/day (0.3g qid) and maximal dose of 2.0g/day (0.5g qid) with an average dose of 1.3g/day for 1 year. B. Amiodarone was taken orally with an initial dose of 0.6g/day (0.2g tid) in the first week, then 0.4g/day (0.2g bid) in the second week and followed by 0.2g/day (0.2g qd) in the third week and for the rest of the year. Follow-up time points were 1-, 3-, 6-, 12-month after treatment.
